# A Rare Encounter: Hepatomegaly Unmasked as Multiple Giant Hepatic Hemangiomas

**DOI:** 10.7759/cureus.68814

**Published:** 2024-09-06

**Authors:** Vasundara Gopalan, Darshana Tote, Anup A Zade, Shubham Durge, Jubin John, Abhilasha Bhargava

**Affiliations:** 1 General Surgery, Jawaharlal Nehru Medical College, Datta Meghe Institute of Higher Education and Research, Wardha, IND; 2 Radiodiagnosis, Jawaharlal Nehru Medical College, Datta Meghe Institute of Higher Education and Research, Wardha, IND

**Keywords:** arterial embolization, giant hemangioma, hemangioma, hepatic angiosarcoma, hepatomegaly

## Abstract

A hepatic hemangioma is a benign liver tumor made up of a number of blood-filled chambers surrounded by liver-supplied endothelial cells. Most liver hemangiomas are asymptomatic and are only discovered during imaging studies for other conditions. Ultrasound is used for initial screening followed by a computed tomography scan, which shows slow enhancement due to small vessels and can be used to diagnose the location, number, and size of a hepatic hemangioma. A large liver hemangioma can range in size from 10 centimeters to more than 20 centimeters and can cause symptoms and complications that require prompt intervention. Hepatic hemangiomas can co-occur with other localized hepatic lesions; a careful diagnosis is necessary to distinguish them. In this case study, a 48-year-old woman complained of a stomachache that had persisted for three months. Following an initial clinical evaluation, hepatomegaly was found, and contrast-enhanced computed tomography (CECT) abdomen and pelvis was performed, revealing numerous giant hepatic hemangiomas. Significant improvements were noted in the patient's condition with tumor embolization.

## Introduction

Hepatic hemangiomas are benign tumors of the liver. A family history of hepatic hemangiomas raises the possibility of a genetic link to mesenchymal genesis. Most of these tumors are asymptomatic, and they are typically found by accident during imaging studies for unrelated illnesses, most are picked incidentally on ultrasonography [1]. A typical computed tomography (CT) lesion showing discontinuous, nodular, peripheral enhancement on the arterial phase with progressive centripetal fill-in on the portal venous, venous, and delayed phases is a well-defined hyperechoic lesion on ultrasound and a hypoattenuating lesion on CT. It is identified by the characteristic appearance of slow contrast enhancement owing to small vessel uptake in the hemangioma [1,2]. Typical hemangiomas range in size from a few millimeters to 3 centimeters, do not grow in size over time, and hence are unlikely to produce symptoms. Small and medium hemangiomas (less than 10 centimeters) are well-defined lesions that do not require active treatment beyond monthly check-ups. However, big hepatic hemangiomas demand immediate intervention [1-3]. Hemangiomas larger than 4-5 centimeters are defined as "giant hemangiomas" and are usually benign and asymptomatic [1,4]. This is a case of a 48-year-old female with abdominal pain for three months, later diagnosed as giant hepatic hemangiomas. The patient was successfully managed by embolization as noted in the follow-up at six months by tumor size reduction.

## Case presentation

**Figure 1:**
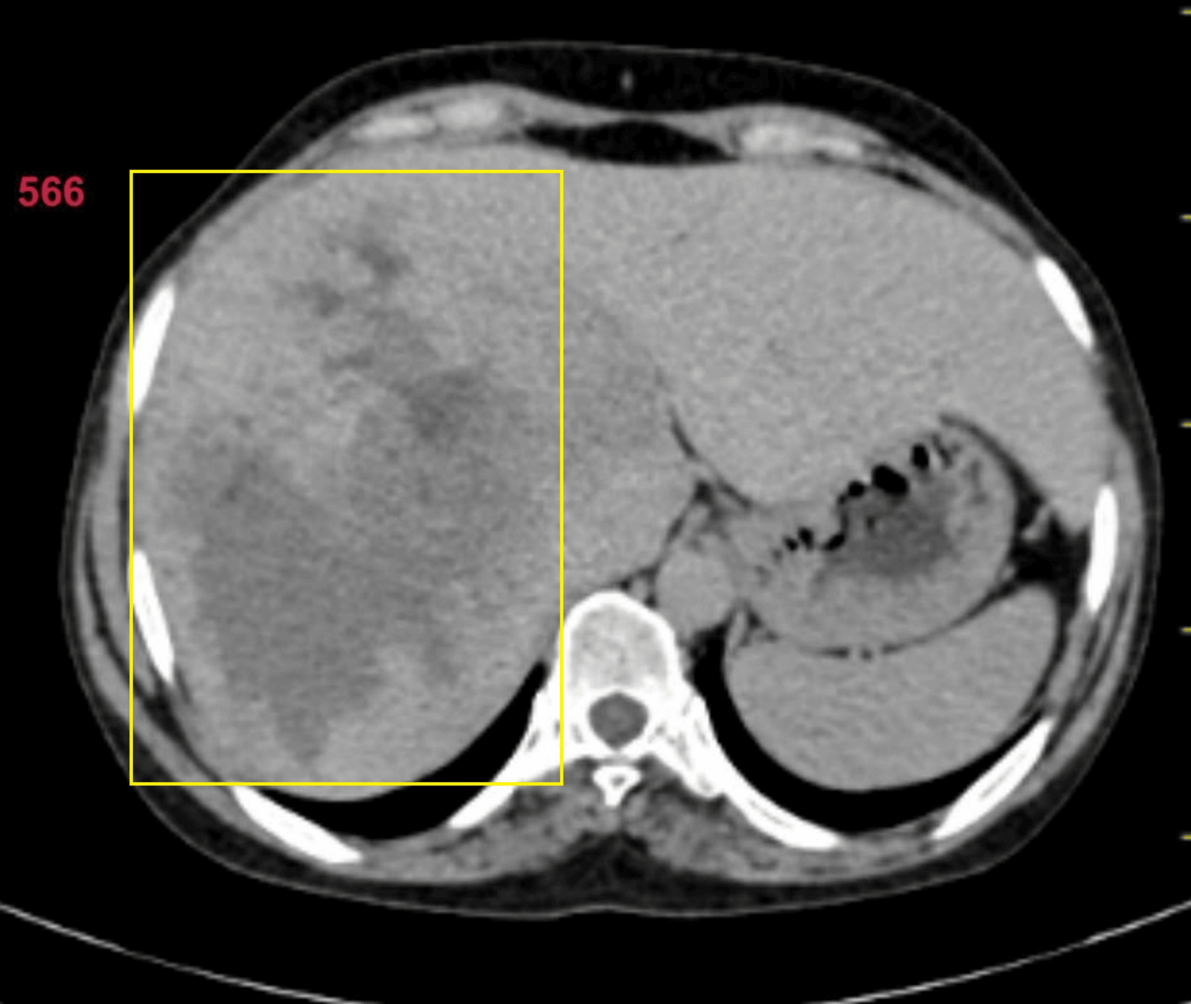
CECT of the pelvis showing hemangioma in the liver CECT: contrast-enhanced computed tomography

A 48-year-old female came with major complaints of pain in the abdomen for three months, insidious in onset, gradually progressing to dull aching, and non-radiating in nature. There was no history of trauma, fever, weight loss, nausea, vomiting, bladder irregularities, or change in bowel habits. The patient neither consumed alcohol nor smoked. The patient did not have a positive history of any major illness or comorbidities. On examination, the patient had hepatomegaly. Laboratory findings of the patient were noted as hemoglobin of 11.8 grams per deciliter, total white blood cell count of 4300 cells per microliter, and international normalized ratio of 1.01, and the rest of the related parameters such as amylase, lipase, liver function tests, and alpha-fetoprotein were found to be within normal limits. The patient underwent contrast-enhanced computed tomography (CECT) abdomen and pelvis, which was suggestive of hepatomegaly with left lobe hypertrophy with two well-defined hypointense lesions in the right lobe of the liver sparing segment six measuring 13.0 x 13.5 x 15.7 centimeters with marked central areas of attenuation (Figure 1).

A similar lesion of size 5.2 x 4.3 x 5.8 centimeters was noted in segment four B of the left lobe of the liver, likely giant hepatic hemangiomas. The patient underwent prophylactic tumor embolization after selective canulation of the celiac artery and selective canulation of the right hepatic artery using a Progreat microcatheter. An angiogram showed a contrast blush in the segment right lobe of the liver (Video 1, Video 2, Video 3).

**Video 1:** Angiogram showing a large hepatic tumor and peripheral puddling of the contrast material

**Video 2:** Angiogram showing embolization using bleomycin/lipiodol combination using a microcatheter

**Video 3:** Final shoot showing good deposition of lipiodol with good embolization of the hemangioma

Embolization was done using bleomycin and 355-500 µm size polyvinyl alcohol foam particles with a completed angiogram demonstrating successful embolization of the tumor. The patient was followed up subsequently after three months with drastic improvement of symptoms. The patient was advised for a follow-up CT scan after six months for the evaluation of the tumor size.

## Discussion

Giant hemangiomas are often classified as hemangiomas that measure more than 4-5 cm. The single-layer endothelial cells that cover the network of capillaries in a microscopically large hemangioma are not enclosed by a capsule but rather clearly separated from the surrounding hepatic parenchyma [2,4]. The giant variation is characterized by an exophytic growth pattern and a high degree of vascularization. Patients who are asymptomatic or whose hemangioma is smaller than 5 cm are monitored without receiving any active treatment [5,6]. Individuals who have hepatic hemangiomas may exhibit symptoms like right hypochondrium pain. Additional symptoms stem from the compression of surrounding organs leading to respiratory difficulties, with obstructive jaundice, and nausea and vomiting in the stomach [2,4]. Active intervention may be necessary in patients who exhibit symptoms or when the lesion is larger than 5 centimeters.

Surgery is only used to treat 2% of hepatic angiomas; however, this number increases to 18-57% in the case of gigantic angiomas. Surgical excision, selective angiographic embolization, selective surgical transhepatic closure of major feeding arteries, and radiofrequency ablation are among the several therapeutic techniques [7]. Segmental resection or enucleation are available surgical options. Postoperative morbidity is low as laparoscopic and robotic operations have grown more common. Large tumors that take up the entire lobe may require a right or left hepatectomy [4,8]. By catheterizing the hepatic arteries, polyvinyl alcohol or other compounds are used to do selective angiographic embolization, which is the result of a reduction in the tumor size [9,10]. When hemangiomas have a clearly defined arterial blood supply, the technique is helpful in limiting and reversing tumor growth [6,9,11]. By inducing tissue necrosis, selective surgical closure of major feeding vessels aids in the tumor’s effective shrinkage.

Another option for ablation is radiofrequency [2,8,9]. Giant hepatic hemangioma is managed surgically, but this case was managed by arterial embolization as also recommended by Tonpe et al., which was found helpful in giant cavernous hemangioma as a non-surgical option in patients that cannot undergo surgical management due to the giant size and associated risks [8,10]. This case was managed successfully by polyvinyl alcohol foam particles, which was evident by the tumor size reduction. Hence, we recommend arterial embolization as a non-surgical management option in patients unable to opt for surgical treatment.

## Conclusions

Hepatic hemangiomas are common and usually resolve without complications. Conservative care is typically suitable for instances that are asymptomatic and not advancing. However, in some cases, hemangiomas occasionally develop to a considerable size, which can cause symptoms and consequences. To alleviate symptoms and avoid serious complications, accurate diagnosis and prompt management are crucial.
